# 100 years of recovery and prognosis in schizophrenia

**DOI:** 10.1192/j.eurpsy.2023.2247

**Published:** 2023-07-19

**Authors:** I.-M. Molstrom, J. Nordgaard, A. Urfer-Parnas, R. Handest, J. Berge, M. G. Henriksen

**Affiliations:** 1 Mental Health Center Amager, University Hospital of Copenhagen; 2 Department of Clinical Medicine, University of Copenhagen, Copenhagen, Denmark; 3 Department of Clinical Sciences, Lund University, Lund, Sweden; 4 Center for Subjectivity Research, University of Copenhagen, Copenhagen, Denmark

## Abstract

**Introduction:**

Recovery in schizophrenia is both widely accepted and commonly misunderstood. Researchers have described favorable outcomes for schizophrenia for the last 100 years. Nevertheless, many patients, relatives and clinicians view schizophrenia as a disease with an inevitable chronic course, as described by Kraepelin in 1889. The definition and measurement of recovery in schizophrenia have proven to be a difficult task. If defined by the remission of clinical symptoms, we have criteria that are operational, but is symptomatic remission sufficient to describe recovery? If looking at social recovery, outcomes related to recovery e.g., social life, employment or social engagement are not easily measured by reliable independent metrics. Thirdly, recovery can be described as a personal journey rather than a clinical endstate.

**Objectives:**

The aim is to present a historical and global overview of 100 years of research in recovery in schizophrenia.

**Methods:**

We conducted a systematic review and meta-analysis. We included prospective studies with at least 20 years of follow-up on patients with a diagnosis of schizophrenia, and the studies must include face-to-face clinical evaluation. We examined outcome in three nested groups: ‘recovery’, ‘good or better’ (i.e., good and recovery), and ‘moderate or better’ (i.e., moderate, good, and recovery). We used random-effects meta-analysis and meta-regression to examine mean estimates and possible moderators.

**Results:**

The overview will start with Bleuler, who described that approximately one third have a good outcome, and end with the most recent meta-analyses on recovery in schizophrenia, presenting both data from our own research and others on the recovery of schizophrenia. Ultimately, we will discuss whether recovery have improved in the last 100 years.

**Conclusions:**

It is a myth that schizophrenia inevitably has a deteriorating course. Recovery is certainly possible. Schizophrenia remains, however, a severe and complex mental disorder, exhibiting a limited change in prognosis despite more than 100 years of research and efforts to improve treatment.

**Disclosure of Interest:**

None Declared

